# Cerebral ^18^F-FDG PET in macrophagic myofasciitis: An individual SVM-based approach

**DOI:** 10.1371/journal.pone.0181152

**Published:** 2017-07-13

**Authors:** Paul Blanc-Durand, Axel Van Der Gucht, Eric Guedj, Mukedaisi Abulizi, Mehdi Aoun-Sebaiti, Lionel Lerman, Antoine Verger, François-Jérôme Authier, Emmanuel Itti

**Affiliations:** 1 Department of Nuclear Medicine, H. Mondor Hospital, Assistance Publique-Hôpitaux de Paris/Paris-Est University, Créteil, France; 2 Department of Nuclear Medicine, La Timone Hospital, Assistance Publique-Hôpitaux de Marseille, Marseille, France; 3 Aix-Marseille University, INT, CNRS UMR 7289, Marseille, France; 4 Aix-Marseille University, CERIMED, Marseille, France; 5 INSERM U955-Team 10, Créteil, France; 6 Department of Neurology, H. Mondor Hospital, Assistance Publique-Hôpitaux de Paris/Paris-Est University, Créteil, France; 7 CHU Nancy, Nuclear Medecine & Nancyclotep Experimental Imaging Platform, Nancy, France; 8 Department of Pathology, H. Mondor Hospital, Assistance Publique-Hôpitaux de Paris/Paris-Est University, Créteil, France; 9 Reference Center for Neuromuscular Disorders, H. Mondor Hospital, Assistance Publique-Hôpitaux de Paris, Créteil, France; 10 INSERM U955-GRC Amyloid Research Institute, Créteil, France; Harbin Institute of Technology Shenzhen Graduate School, CHINA

## Abstract

**Introduction:**

Macrophagic myofasciitis (MMF) is an emerging condition with highly specific myopathological alterations. A peculiar spatial pattern of a cerebral glucose hypometabolism involving occipito-temporal cortex and cerebellum have been reported in patients with MMF; however, the full pattern is not systematically present in routine interpretation of scans, and with varying degrees of severity depending on the cognitive profile of patients. Aim was to generate and evaluate a support vector machine (SVM) procedure to classify patients between healthy or MMF ^18^F-FDG brain profiles.

**Methods:**

^18^F-FDG PET brain images of 119 patients with MMF and 64 healthy subjects were retrospectively analyzed. The whole-population was divided into two groups; a training set (100 MMF, 44 healthy subjects) and a testing set (19 MMF, 20 healthy subjects). Dimensionality reduction was performed using a t-map from statistical parametric mapping (SPM) and a SVM with a linear kernel was trained on the training set. To evaluate the performance of the SVM classifier, values of sensitivity (Se), specificity (Sp), positive predictive value (PPV), negative predictive value (NPV) and accuracy (Acc) were calculated.

**Results:**

The SPM12 analysis on the training set exhibited the already reported hypometabolism pattern involving occipito-temporal and fronto-parietal cortices, limbic system and cerebellum. The SVM procedure, based on the t-test mask generated from the training set, correctly classified MMF patients of the testing set with following Se, Sp, PPV, NPV and Acc: 89%, 85%, 85%, 89%, and 87%.

**Conclusion:**

We developed an original and individual approach including a SVM to classify patients between healthy or MMF metabolic brain profiles using ^18^F-FDG-PET. Machine learning algorithms are promising for computer-aided diagnosis but will need further validation in prospective cohorts.

## Introduction

Macrophagic myofasciitis (MMF) is an emerging condition with highly specific myopathological alterations found at deltoid muscle biopsy assessing persistence of aluminum hydroxide adjuvant particles within macrophages that may occur following intramuscular vaccine injections (#ORPHA592, http://www.orpha.net) [1,2]. In most patients, clinical manifestations typically associated with MMF include arthromyalgias, chronic fatigue and cognitive impairment, occurring several months or years after the last vaccine injection [3–5]. Few functional SPECT and PET studies have investigated this cognitive disorder [6–8]. A peculiar spatial pattern of a cerebral glucose hypometabolism involving occipito-temporal cortex and cerebellum have been reported [7]; however, the full pattern is not systematically present in routine interpretation of scans, and with varying degrees of severity depending on the cognitive profile of patients [8]. Thus, the identification of an individual biomarker is needed.

For this issue, computer aided diagnosis has already shown to be more efficient than physicians for special task but is also known to produce lots of false positives. Therefore, it is needed to use validated techniques that refer to the term “decoding”. In terms of neuroimaging, decoding refers to learning parameters from brain imaging data (hence would represent voxels values) to predict an outcome, here the disease status that has a binary output and hence represent a supervised classification learning problematic. Two main approaches for computer aided diagnosis are currently used, one using handcrafted features such as shape features [9], textural features [10] or wavelet transform. The second approach uses high-level features automatically built by algorithms that are then taught to an algorithm to distinguish between disease and healthy conditions. When the number of parameters is higher than the number of training samples, some machine learning techniques can be applied such as random forest, least absolute shrinkage and selection operator (LASSO) logistic regressions [11], or support vector machines (SVM) [12].

Applied to ^18^F-FDG PET brain imaging, SVM classifier recently showed high accuracy to distinguish patients with amyotrophic lateral sclerosis from controls and assess individual prognosis [13]. Indeed, SVM method is a supervised classification machine-learning algorithm commonly used in neuroimaging for multi-voxel pattern analysis either for PET [13] or MRI [14]. SVM classifiers are also more and more used in various bioinformatics fields and at different scales such as dicer cleavage sites prediction [15] for miRNA, protein homology detection [16], and in biomedical imaging for example for decoding [17]. Briefly, from a training set of diseased patients versus normal controls, the most discriminant voxels must be pre-selected to avoid the so-called curse of dimensionality. Many approaches can be performed for that, first base on neuroscientific knowledge or in a second approach feature selection that rely on univariate statistical testing such as t-test or ANOVA. On the reduced feature voxel dataset, a SVM is trained and coefficients are identified and then applied to another set of patients and controls to determine the diagnostic performance. Then, the aim was to generate and evaluate a SVM procedure to classify patients between healthy or MMF ^18^F-FDG brain profiles.

## Materials and methods

### MMF patients

The study population included consecutive symptomatic patients with histopathological features of MMF at muscle biopsy. Patients with a history of cerebral disease were excluded. All patients underwent brain ^18^F-FDG PET/CT as standard care. The Institutional Review Board (Comité de Protection des Personnes Ile-de-France VI) took into account the retrospective nature of this study and approved the protocol (December 18, 2013).

### Healthy subjects

Healthy controls from the NCT00484523 clinical trial matched for age were included. They were free from neurological/psychiatric disease and cognitive complaints, had a normal brain MRI, and underwent a PET/CT scan prior to a standardized neurological examination including a Mini Mental State Examination to check the lack of cognitive impairment. Due to a limited number of healthy patients in our institution, the healthy patients included in the current study came from another centre (Marseille). Using SPM analysis, no significant difference in FDG uptake was found between ^18^F-FDG PET brain images of these patients and images of 5 healthy subjects of our institution.

### FDG-PET/CT acquisition

As previously described [8], brain imaging of MMF patients was performed on a Gemini GXL PET/CT camera (Philips, Da Best, The Netherlands) after intravenous injection of 2 MBq/kg ^18^F-FDG. Patients were required to fast for at least 6 h before undergoing the scan, had normal blood glucose levels and were maintained in neurosensory resting 10 min before and for 30 min after injection. Brain imaging of healthy controls had been performed on a Discovery ST PET/CT camera (General Electric, Milwaukee, WI), using similar acquisition parameters (1 step of 15 min, 30 min after intravenous injection of 150 MBq ^18^F-FDG).

### Statistical parametric mapping analysis

**Fig 1:**
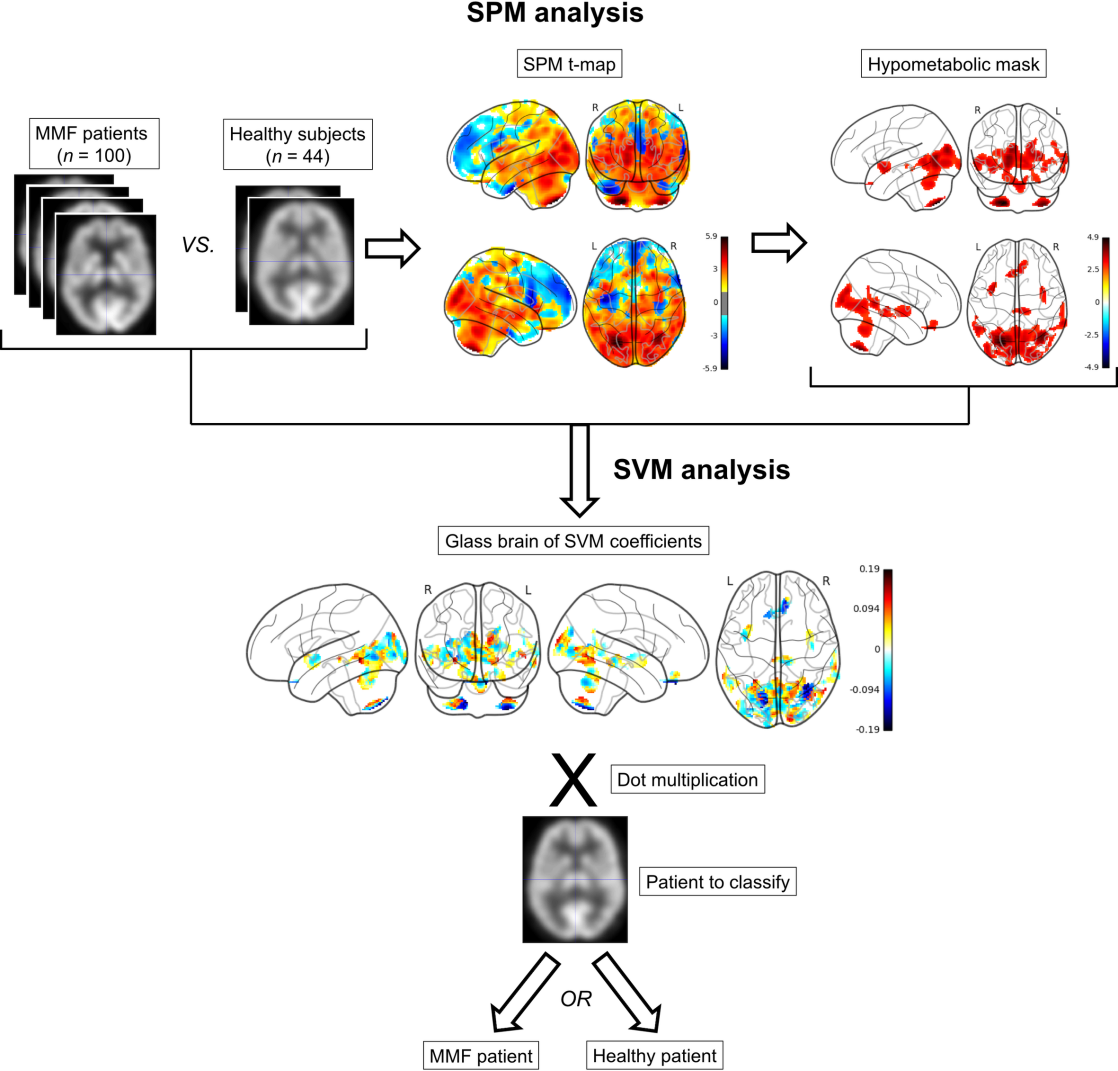
Statistical parametric mapping (SPM) and support vector machine (SVM) procedures. MMF, Macrophagic myofasciitis; L, left; R, right.

All ^18^F-FDG-PET brain image volumes were spatially normalized onto the Montreal Neurological Institute template (McGill University, Montreal, Canada). Dimensions of the resulting voxels were 2x2x2 mm^3^. Images were smoothed using a Gaussian filter (FWHM 8 mm). Spatial preprocessing and statistical analysis were performed using the statistical parametric mapping (SMP12) software implemented in Matlab version R2015a (Mathworks Inc., Sherborn, MA). The population of our previous report [8] (100 MMF patients, 44 healthy subjects) was used to train the SVM classifier. Thirty-nine additional subjects (19 MMF patients and 20 healthy subjects) served as a testing population. As shown in Fig 1, using analysis of covariance (ANCOVA) in SPM12 with adjustment for age, a t-test mask was generated from the comparison between cerebral ^18^F-FDG PET images of MMF patients and healthy subjects. As used in our previous report [8], results of the comparison were collected at a P-value < 0.005 at the voxel level, for clusters k ≥ 200 voxels (corrected for cluster volume). The t-test aimed to reduce the number of voxels to include in the model, keeping only those which were statistically different between the two groups.

### Support vector machine classifier

On the reduced feature set when considering only the significant voxels provided with SPM, a SVM classifier was trained on the training population set with a linear kernel. SVM, in the multidimensional space of the *N* features, searched the best hyperplane with the maximum margin, which means that the separation of the two classes is maximized by the choice of the hyperplane. The resulting coefficients (SVM discriminative weights) were represented as a glass brain in Fig 1. They represent the weights that, when dot multiplicated with brain PET values, allow to differentiate between pathologic or healthy. All these computations were performed using python 2.7 with nilearn and scikit-learn [18] packages. To assess the performance of the SVM classifier, values of sensitivity (Se), specificity (Sp), positive predictive value (PPV), negative predictive value (NPV) and accuracy (Acc) were calculated on the testing set of 39 patients. Se, Sp, PPV and NPV and Acc were defined as follow:

$$Se=\frac{TP}{TP+FN}$$

$$Sp=\frac{TN}{TN+FP}$$

$$PPV=\frac{TP}{TP+FP}$$

$$NPV=\frac{TN}{TN+FN}$$

$$Acc=\frac{TP+TN}{TP+FP+TN+FN}$$

where TP (True Positive) is the number of MMF patients correctly classified as such by the SVM approach, TN (True Negative) is the number of healthy patients correctly classified as such, FP (False Positive) is the number of healthy patients wrongly classified as MMF and FN (False Negative) is the number of MMF patients wrongly classified as healthy.

## Results

### Population characteristics

^18^F-FDG PET brain images of 119 patients with MMF and 64 healthy subjects were retrospectively analyzed. The whole-population was divided into two groups; a training set with 100 MMF and 44 healthy subjects (mean age: 46 ± 14 y) recently published [8], and a new testing set with 19 additional MMF patients and 20 additional healthy subjects (mean age: 52 ± 15 y). Population characteristics for the training and testing groups are given in Table 1. All relevant data are provided in S1 Table.

**Table 1:** Population characteristics for the training and testing groups.

Characteristics	Training population		Testing population	
	MMF patients	Healthy subjects	MMF patients	Healthy subjects
N	100	44	19	20
Age (years)	46.5 ± 12	45.4 ± 16	42.5 ± 15	52 ± 15
Gender				
Male	25	12	4	6
Female	75	32	15	14
Diffuse arthromyalgias	94 (94)	NA	15 (79)	NA
Chronic fatigue	69 (69)	NA	11 (58)	NA
Cognitive impairment	76 (76)	NA	13 (68)	NA

Values are mean ± SD or n (%)

### SPM analysis and SVM classifier

**Fig 2:**
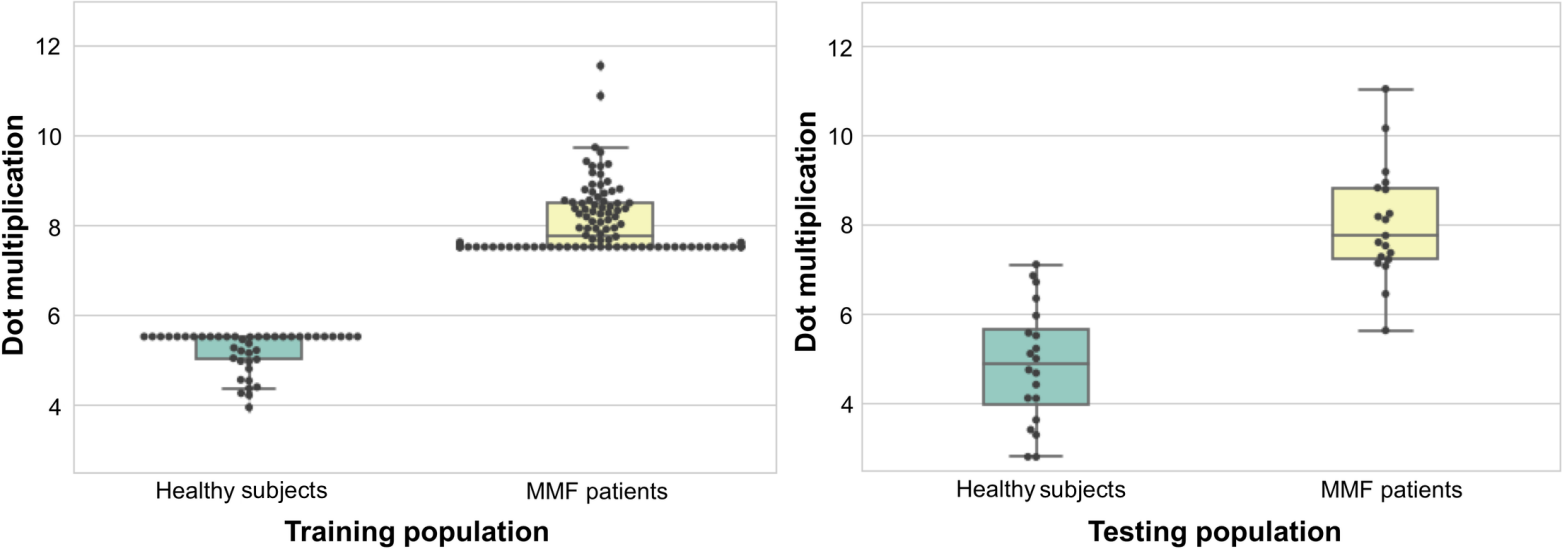
Box plots of the dot multiplication in training and testing populations.

Using the training population, SPM12 analysis exhibited the already reported hypometabolic pattern involving occipito-temporal and fronto-parietal cortices, limbic system and cerebellum [8]. These areas were the most discriminating brain regions (Fig 1 and Table 2). On the testing population, with 2 false negatives and 3 false positives (Table 3), the SVM correctly classified into the MMF pattern with Se, Sp, PPV, NPV and Acc of 89.4%, 85.0%, 85.0%, 89.4% and 87.2%, respectively. This performance can be seen on the boxplot representing the result of the dot multiplications of the coefficients and the normalized ^18^F-FDG-PET intensity values of each patient and each voxel. Indeed, on the training set, we can see that the dot multiplication can clearly differentiate between MMF and healthy patients (Fig 2). This net separation is partly lost on the validation set of 39 patients but remains statistically significant (Mann Whitney U test p-value <0.01) and is the reason of 2 false negatives and 3 false positives.

**Table 2:** Comparison between ^18^F-FDG PET brain images of 100 MMF patients and 44 healthy subjects included in the training set. Brain areas with significant decreased uptake of ^18^F-FDG served as mask to train the support vector machine classifier. Results were collected at a P-value < 0.005 at the voxel level, for clusters k ≥ 200 voxels with adjustment for age.

K	Brain areas	Side	Labels	Peak value coordinates (mm)			T-value	P-value
				*x*	*y*	*z*		
367	Cerebellum	L	CPL	-22	-72	-60	4.91	<0.001
326	Cerebellum	R	CPL	36	-66	-62	4.33	<0.001
5379	Occipital lobe, Cerebellum, Limbic lobe, Temporal lobe, Sublobar region, Parietal lobe	L, R	BA18-BA19-BA17-BA30-BA23-BA37-BA31-BA7-CAL-CPL	-12	-70	6	4.10	<0.001
417	Temporal lobe, Parietal lobe	R	BA21-BA22-BA40-BA39	66	-46	4	3.57	<0.001
264	Sublobar region, Temporal lobe	L	BA13-BA38	-36	4	-8	3.54	<0.001
427	Temporal lobe, Occipital lobe	L	BA37-BBA39-BA21-BA19-BA22	-56	-54	0	3.53	<0.001
200	Frontal lobe	L, R	BA11-BA47	-8	22	-32	3.45	<0.001
207	Midbrain, Sub-lobar, Limbic lobe	R	BA27-BA28-BA30-BA35	12	-30	-6	3.32	0.001
271	Sublobar region, Temporal lobe	R	BA13-BA21	40	-2	-8	3.16	0.001

BA, brodmann area; CAL, cerebellum anterior lobe; CPL, cerebellum posterior lobe; L, left; R, right

**Table 3:** Confusion matrix of the result of the support vector machine classifier for the diagnosis.

	Patients classified as MMF	Patients classified as healthy	Total
MMF patients	17	2	19
Healthy patients	3	17	20
Total	20	19	39

**Fig 3:**
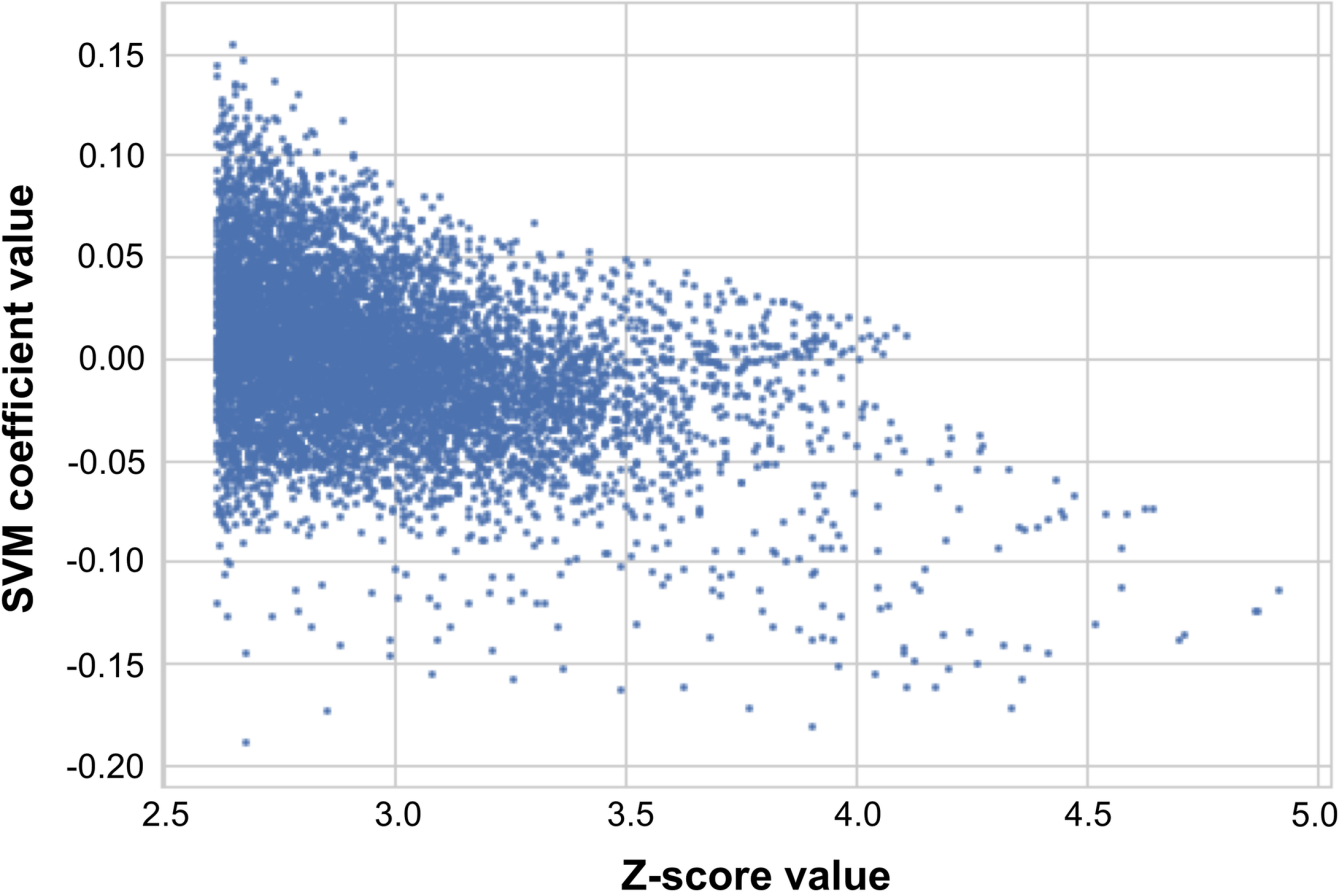
Scatter plot of the coefficient value from the support vector machine (SVM) as a function of the Z-score from statistical parametric mapping (SPM).

As shown in Fig 3, the distribution of the coefficients weights did not show any preferential distribution neither through the brain classically affected by MMF nor within regional distribution between cortical or subcortical areas. Nevertheless, as coefficients with higher values have more influence on the prediction than the others, we performed a scatter plot of the coefficients of the SVM as a function of the Z-score voxel value. It revealed a threshold without linear tendency. Indeed, the higher the Z-score voxel intensity value is, the lower and negative the coefficient.

## Discussion

The aim of the current study was to generate and evaluate a support vector machine procedure to classify patients between healthy or MMF ^18^F-FDG brain profiles. The generated t-test mask was that of our previous findings [8], corresponding to a symmetrical pattern of hypometabolism involving occipital, temporal lobes but also the limbic system, fronto-paritetal cortex and both hemispheric cerebellum. Nevertheless, these analyses were performed at the scale of a group where the signal was smoothed. An ANCOVA was performed between the two groups of healthy or MMF patients which aims to compare the means of each voxels, and how they statistically differ with additional qualitative covariate (age). Here, we present an original procedure where we build a SVM classifier at the patient level to classify each patient between healthy or pathologic brain profiles. Other classifiers could have been used such as artificial neural networks, random forest classifiers or Bayesian techniques. Artificial neural networks or multilayer perceptron have the ability to learn nonlinear models by back-propagating errors [19]. However, they require bigger populations to train and have non-convex cost function that may not always converge to the same values. Similarly, random forest classifiers (which are random tree classifiers based on bootstrapping) may not give identical results each time the algorithm is run. Inversely, the SVM approach has the advantage to be easily implemented, fast to train and works well when the number of parameters is higher than the number of patients.

The resulting coefficients seem linked with the Z-score value with a threshold limit. The Z-score value at a voxel level represent the number of standard deviation from which mean voxels of MMF patients differs from the mean of healthy patients. The higher the Z-score is, the more different this voxel was between healthy and the MMF patients. As we specifically seek the hypometabolism of MMF patients, the higher Z-score represent voxels that are particularly affected in MMF with much lower means. As shown in Fig 3, patients with MMF had a dot multiplication (between coefficient and their PET intensity value) that was higher than the ones of healthy patients. This might seem counter-intuitive but is explained by the fact that as the MMF patients have lower voxel intensity values, their dot multiplication by the negative coefficients of the SVM and their PET intensity value is less affected than the healthy patients as they have higher voxel intensity values. On a testing set of 39 patients (who were therefore not included in the training set) satisfactory results of diagnostic performance were obtained but could be overestimated in everyday practice as it was evaluated only in patients with MMF against healthy controls. This approach, as they classify patient between healthy or pathologic may also be interesting to follow patients. Even if MMF cognitive dysfunction seems to not worsen nor ameliorate over time based on neuropsychological assessment [20] (which was also observed with ^18^F-FDG PET [21]); nevertheless, some therapies may be some day able to reverse the brain abnormality profile or prevent it to appear which could be measured with ^18^F-FDG PET and this kind of approach.

Main perspectives include validation of this classifier in larger and multicentric cohorts in prospective trials, but could also help to better characterize other patients with other similar conditions like fibromyalgias (FM) which share common symptoms such as myalgias or fatigue. As there are no pathognomonic findings in FM, diagnosis is made on criteria that have been defined by the American college of rheumatology [22]. Indeed FM induces brain perfusion abnormalities when compared to healthy controls on ^99m^Tc-ECD SPECT [23] or ^18^F-FDG PET [24] studies with hyperperfusion of the somatosensory cortex and occipital lobe where as a relative hypoperfusion of the frontal, cingulate, temporal and cerebellum that are different from those observed in MMF patients. Furthermore, as ^18^F-FDG PET scans of FM patients when compared to healthy controls revealed no statistical difference [25], we hypothesize that the brain abnormalities of MMF and FM may be different. Hence some patients who were classified by error as FM patients could maybe reclassified as MMF patients based on their ^18^F-FDG-PET brain hypometabolic profile. Computer-aided diagnostic is an interdisciplinary field combining elements of computer vision and machine learning. It has been already implemented in clinical practice for example in pulmonary nodule detection [26] or classification for cytopathology [27].

The main limits of this report are the retrospective nature of this study, and that all the healthy subjects, either in the testing or training groups, are from a different institution than that the MMF patients come from. Therefore, even if all patients were rescaled to a MNI template and normalized, observed differences may be influenced from the differences between image acquisition and reconstruction protocols. Nevertheless, as mentioned in the methodology part, we first checked that our healthy patients didn’t statistically differ from the healthy patients of the other institution. Furthermore, we also checked that the 5 healthy subjects from Henri Mondor Hospital were accurately classified as healthy.

## Conclusion

In conclusion, the SVM procedure appears as a useful individual tool to classify subjects between healthy or MMF ^18^F-FDG PET brain profiles. We developed an original approach with first a features reduction using Z-score maps with SPM analysis. Secondly, an SVM procedure was trained and coefficients weights were studied as they seem linked to the Z-score value. Machine learning algorithms are promising for computer-aided diagnosis either for classification or regression problematics. As they will gain more and more importance in the year to come, they will challenge traditional interpretation of examination but surely will help physicians and healthcare providers more comprehensive insights on diverse pathologies.

## Supporting information

S1 Table All relevant data. (DOC)
