# Supplementary material for: Cerebral 18F-FDG PET in macrophagic myofasciitis: An individual SVM-based approach
Source: PLoS One. 2017 Jul 13;12(7):e0181152. doi: 10.1371/journal.pone.0181152 (PMC5509294; doi:10.1371/journal.pone.0181152)
Supplement: S1 Table — (DOC) [file pone.0181152.s001.doc]

**Table S1**. All relevant data.

| **Population** | **Patient** | **Gender**  **(1 = woman)** | **Diffuse arthromyalgias (1 = Yes)** | **Chronic fatigue**  **(1 = Yes)** | **Cognitive impairment (1 = Yes)** |
| --- | --- | --- | --- | --- | --- |
| MMF Traing Population | 1 | 1 | 1 | 1 | 1 |
| MMF Traing Population | 2 | 0 | 1 | 1 | 1 |
| MMF Traing Population | 3 | 1 | 1 | 1 | 1 |
| MMF Traing Population | 4 | 1 | 1 | 1 | 1 |
| MMF Traing Population | 5 | 0 | 0 | 0 | 0 |
| MMF Traing Population | 6 | 1 | 0 | 0 | 1 |
| MMF Traing Population | 7 | 0 | 1 | 1 | 1 |
| MMF Traing Population | 8 | 1 | 1 | 1 | 1 |
| MMF Traing Population | 9 | 1 | 0 | 0 | 0 |
| MMF Traing Population | 10 | 1 | 1 | 1 | 1 |
| MMF Traing Population | 11 | 1 | 1 | 1 | 1 |
| MMF Traing Population | 12 | 0 | 0 | 1 | 1 |
| MMF Traing Population | 13 | 1 | 1 | 0 | 0 |
| MMF Traing Population | 14 | 1 | 1 | 0 | 1 |
| MMF Traing Population | 15 | 0 | 1 | 1 | 1 |
| MMF Traing Population | 16 | 0 | 1 | 0 | 1 |
| MMF Traing Population | 17 | 1 | 1 | 1 | 0 |
| MMF Traing Population | 18 | 0 | 1 | 1 | 1 |
| MMF Traing Population | 19 | 0 | 1 | 1 | 1 |
| MMF Traing Population | 20 | 1 | 1 | 0 | 1 |
| MMF Traing Population | 21 | 1 | 1 | 1 | 1 |
| MMF Traing Population | 22 | 0 | 1 | 0 | 0 |
| MMF Traing Population | 23 | 1 | 1 | 0 | 1 |
| MMF Traing Population | 24 | 1 | 1 | 1 | 1 |
| MMF Traing Population | 25 | 1 | 1 | 1 | 1 |
| MMF Traing Population | 26 | 1 | 1 | 0 | 0 |
| MMF Traing Population | 27 | 1 | 1 | 1 | 0 |
| MMF Traing Population | 28 | 1 | 1 | 0 | 1 |
| MMF Traing Population | 29 | 0 | 1 | 1 | 1 |
| MMF Traing Population | 30 | 1 | 1 | 1 | 1 |
| MMF Traing Population | 31 | 1 | 1 | 1 | 1 |
| MMF Traing Population | 32 | 0 | 1 | 1 | 1 |
| MMF Traing Population | 33 | 1 | 1 | 0 | 1 |
| MMF Traing Population | 34 | 1 | 1 | 0 | 0 |
| MMF Traing Population | 35 | 1 | 1 | 1 | 1 |
| MMF Traing Population | 36 | 1 | 1 | 1 | 1 |
| MMF Traing Population | 37 | 1 | 1 | 0 | 0 |
| MMF Traing Population | 38 | 1 | 1 | 1 | 1 |
| MMF Traing Population | 39 | 1 | 1 | 1 | 1 |
| MMF Traing Population | 40 | 0 | 0 | 1 | 1 |
| MMF Traing Population | 41 | 1 | 1 | 1 | 1 |
| MMF Traing Population | 42 | 0 | 1 | 0 | 0 |
| MMF Traing Population | 43 | 1 | 1 | 0 | 0 |
| MMF Traing Population | 44 | 0 | 1 | 1 | 0 |
| MMF Traing Population | 45 | 1 | 1 | 1 | 0 |
| MMF Traing Population | 46 | 0 | 1 | 0 | 0 |
| MMF Traing Population | 47 | 1 | 1 | 1 | 1 |
| MMF Traing Population | 48 | 0 | 1 | 1 | 1 |
| MMF Traing Population | 49 | 1 | 1 | 1 | 1 |
| MMF Traing Population | 50 | 0 | 1 | 0 | 1 |
| MMF Traing Population | 51 | 1 | 1 | 1 | 1 |
| MMF Traing Population | 52 | 1 | 1 | 1 | 1 |
| MMF Traing Population | 53 | 1 | 1 | 1 | 1 |
| MMF Traing Population | 54 | 0 | 0 | 1 | 1 |
| MMF Traing Population | 55 | 1 | 1 | 1 | 1 |
| MMF Traing Population | 56 | 1 | 1 | 1 | 1 |
| MMF Traing Population | 57 | 1 | 1 | 1 | 0 |
| MMF Traing Population | 58 | 0 | 1 | 1 | 1 |
| MMF Traing Population | 59 | 1 | 1 | 1 | 1 |
| MMF Traing Population | 60 | 1 | 1 | 1 | 1 |
| MMF Traing Population | 61 | 1 | 1 | 1 | 1 |
| MMF Traing Population | 62 | 1 | 1 | 0 | 0 |
| MMF Traing Population | 63 | 1 | 1 | 0 | 1 |
| MMF Traing Population | 64 | 1 | 1 | 0 | 0 |
| MMF Traing Population | 65 | 1 | 1 | 1 | 0 |
| MMF Traing Population | 66 | 1 | 1 | 0 | 1 |
| MMF Traing Population | 67 | 1 | 1 | 0 | 1 |
| MMF Traing Population | 68 | 1 | 1 | 1 | 1 |
| MMF Traing Population | 69 | 1 | 1 | 1 | 1 |
| MMF Traing Population | 70 | 1 | 1 | 0 | 1 |
| MMF Traing Population | 71 | 1 | 1 | 1 | 1 |
| MMF Traing Population | 72 | 1 | 1 | 1 | 1 |
| MMF Traing Population | 73 | 0 | 1 | 1 | 1 |
| MMF Traing Population | 74 | 1 | 1 | 1 | 1 |
| MMF Traing Population | 75 | 1 | 1 | 1 | 1 |
| MMF Traing Population | 76 | 1 | 1 | 1 | 1 |
| MMF Traing Population | 77 | 0 | 1 | 1 | 1 |
| MMF Traing Population | 78 | 1 | 1 | 1 | 1 |
| MMF Traing Population | 79 | 0 | 1 | 0 | 0 |
| MMF Traing Population | 80 | 1 | 1 | 0 | 0 |
| MMF Traing Population | 81 | 1 | 1 | 1 | 1 |
| MMF Traing Population | 82 | 1 | 1 | 0 | 1 |
| MMF Traing Population | 83 | 0 | 1 | 1 | 1 |
| MMF Traing Population | 84 | 1 | 1 | 0 | 1 |
| MMF Traing Population | 85 | 1 | 1 | 1 | 1 |
| MMF Traing Population | 86 | 1 | 1 | 1 | 1 |
| MMF Traing Population | 87 | 1 | 1 | 0 | 1 |
| MMF Traing Population | 88 | 0 | 1 | 1 | 1 |
| MMF Traing Population | 89 | 1 | 1 | 1 | 1 |
| MMF Traing Population | 90 | 1 | 1 | 1 | 1 |
| MMF Traing Population | 91 | 1 | 1 | 1 | 0 |
| MMF Traing Population | 92 | 0 | 1 | 1 | 1 |
| MMF Traing Population | 93 | 1 | 1 | 1 | 1 |
| MMF Traing Population | 94 | 1 | 1 | 0 | 1 |
| MMF Traing Population | 95 | 1 | 1 | 1 | 1 |
| MMF Traing Population | 96 | 1 | 1 | 1 | 0 |
| MMF Traing Population | 97 | 1 | 1 | 1 | 1 |
| MMF Traing Population | 98 | 1 | 1 | 0 | 0 |
| MMF Traing Population | 99 | 1 | 1 | 1 | 1 |
| MMF Traing Population | 100 | 1 | 1 | 1 | 0 |
| MMF Testing Population | 1 | 1 | 1 | 1 | 1 |
| MMF Testing Population | 2 | 1 | 1 | 1 | 1 |
| MMF Testing Population | 3 | 1 | 1 | 0 | 1 |
| MMF Testing Population | 4 | 1 | 0 | 1 | 0 |
| MMF Testing Population | 5 | 1 | 0 | 0 | 1 |
| MMF Testing Population | 6 | 1 | 1 | 1 | 1 |
| MMF Testing Population | 7 | 1 | 1 | 0 | 0 |
| MMF Testing Population | 8 | 0 | 1 | 0 | 1 |
| MMF Testing Population | 9 | 1 | 1 | 1 | 1 |
| MMF Testing Population | 10 | 0 | 1 | 0 | 0 |
| MMF Testing Population | 11 | 0 | 1 | 1 | 1 |
| MMF Testing Population | 12 | 1 | 1 | 1 | 1 |
| MMF Testing Population | 13 | 1 | 1 | 0 | 0 |
| MMF Testing Population | 14 | 0 | 1 | 1 | 1 |
| MMF Testing Population | 15 | 1 | 1 | 1 | 0 |
| MMF Testing Population | 16 | 1 | 1 | 1 | 1 |
| MMF Testing Population | 17 | 1 | 0 | 0 | 1 |
| MMF Testing Population | 18 | 1 | 0 | 0 | 1 |
| MMF Testing Population | 19 | 1 | 1 | 1 | 0 |
